# Application of HTS for Routine Plant Virus Diagnostics: State of the Art and Challenges

**DOI:** 10.3389/fpls.2018.01082

**Published:** 2018-08-27

**Authors:** Hans J. Maree, Adrian Fox, Maher Al Rwahnih, Neil Boonham, Thierry Candresse

**Affiliations:** ^1^Department of Genetics, Stellenbosch University, Stellenbosch, South Africa; ^2^Agricultural Research Council, Infruitec-Nietvoorbij, The Fruit, Vine and Wine Institute, Stellenbosch, South Africa; ^3^Department of Plant Protection, Fera Science Ltd., York, United Kingdom; ^4^Department of Plant Pathology, University of California, Davis, Davis, CA, United States; ^5^School of Natural and Environmental Sciences, University of Newcastle, Newcastle Upon Tyne, United Kingdom; ^6^UMR 1332 Biologie du Fruit et Pathologie, INRA, University of Bordeaux, Bordeaux, France

**Keywords:** high-throughput sequencing, plant virus detection, validation of diagnostic tool, next generation sequencing (NGS), HTS for virus detection, considerations for HTS detection assays

## Introduction

The application of High-Throughput Sequencing (HTS), also known as next-generation sequencing, has proven very successful for virus discovery to resolve disease etiology in many agricultural crops. Building on this success, the movement to apply HTS for routine virus detection is gaining momentum. Deployment of HTS as a detection tool comes with the same challenges faced when adopting any new method but with some additional technology-specific issues that are explored here.

## HTS for virus detection

The application of HTS for virus discovery has been very successful, in part because it has largely been used in the early stages of a diagnostic investigation, to identify putative viral sequences. From that point onwards more familiar techniques such as PCR or ELISA could be used to provide a definitive diagnosis (Massart et al., 2017). For use in front line detection an unambiguous result is however required to avoid, where possible, multiphasic confirmatory testing. As for any detection assay, attention needs to be paid to aspects of validation such as sensitivity, specificity, reproducibility and repeatability. In establishing these performance criteria it is also key to consider the scenario in which the diagnostic method will be deployed.

### Advantages of HTS for virus detection

The greatest advantage of HTS over other diagnostic approaches is that it gives a complete view of the viral phytosanitary status of a plant. In theory, HTS can detect all viruses in a single assay and performance is limited only by the completeness of the reference database(s) against which the sequences are compared. Sequence information obtained can also be used to provide insight on the virus population structure, ecology or evolution or to differentiate virus variants that may contribute differently toward disease etiology. HTS has the potential to reduce the time from virus discovery to development of targeted detection assays such as PCR or LAMP and to contribute to the improvement of existing assays, by elucidating sequence variation within virus populations. Another advantage of HTS is that sequence data can be analyzed by multiple end-users or may be re-analyzed as databases are expanded.

For the production of propagation stocks, candidate nuclear plants are tested for a range of “targeted or regulated” viruses using a panel of specific methods, such as ELISA, RT-PCR and RT-qPCR, and bioassays (Golino et al., 2017). This involves performing a number of individual tests, which can be challenging for highly variable viruses for which it may be difficult to design a “universal assay” that detects all known and unknown variants. By contrast, HTS is a comprehensive single test that can detect all viruses, including novel variants (Al Rwahnih et al., 2015; Rott et al., 2017). HTS may be more cost effective than a panel of multiple conventional tests. In addition, conventional woody host indexing requires a minimum time of 2–3 years while with HTS the total testing time is 1–2 months (Al Rwahnih, unpublished data). In principle, plant material where no viruses were detected by HTS could be provisionally released, allowing for propagation to start much earlier, with the final release subject to additional testing if required.

### Challenges of HTS for virus detection

In applying HTS for the detection of known viruses there remain both technical and biological challenges. The technical challenge lies in the validation of the technology for the robust detection of a broad range of virus/hosts combinations and in determining the comparability of different approaches for acceptance in routine screening. Validation is analogous to that for other molecular diagnostic assays, as detailed in EPPO PM7/98 (OEPP/EPPO, 2014), with key specific considerations. If used as stand-alone for routine detection, this would mean validating the method against each anticipated virus. However, if a positive detection would lead to confirmatory diagnostics, validation could be focused on minimizing the risk of false negative results (Roenhorst et al., 2018). Routine testing relies upon set processes which have been validated to ascertain their performance characteristics. The rapid pace of development of sequencing platforms, protocols and bioinformatics pipelines brings additional challenges since all improvements may require frequent revalidation to ensure comparable performance.

## Considerations for an HTS detection assay

### Sensitivity

Determining the sensitivity of a method is key when considering the application of a particular diagnostic technique. In the case of HTS approaches, two aspects have to be considered. The first is the intrinsic sensitivity of HTS-based diagnostics. The second concerns the ability of the bioinformatics procedure to detect viral reads among the sequences generated from a sample. Assuming a perfect performance of the bioinformatic analysis, sensitivity is directly linked to the proportion of viral RNAs among the cellular RNAs of the sample, to the efficiency of the enrichment strategy (if one is used) and to the sequencing depth.

In the diagnostics field, novel methods may be validated through direct comparisons with existing, validated methodologies. There have been so far few direct comparisons with HTS approaches and plant viruses. Comparisons with RT-PCR (Rolland et al., 2017) or molecular hybridization (Hagen et al., 2012) suggest a comparable ability to detect viruses in infected samples, however, this may not translate into similar limits of detection. Comparisons with RT-qPCR have shown that HTS has a similar level of sensitivity for the detection of several potato viruses and demonstrated the contribution of the bioinformatics approach, since targeted analysis by mapping reads improved the sensitivity 10-fold (Santala and Valkonen, 2018).

In the case of biological indexing, sometimes considered the gold standard in woody host plants, two large studies in grapevine (Al Rwahnih et al., 2015) and in temperate fruit trees (Rott et al., 2017) also suggest comparable sensitivities. Further comparative efforts, including ones aimed at a determination of limits of detection (Bukowska-Ośko et al., 2017) are clearly needed to clarify the picture on the analytical sensitivity of HTS approaches.

### Specificity

Specificity is an important criterion in the adoption of any diagnostic technique. Because the identification of an agent is based on sequence data, specificity of HTS-based diagnostics is expected to be more predictable and less prone to unexplained cross reactions or false negative results caused by unexpected interactions of reagents with target or host nucleic acids or proteins. Unlike other methods, where the specificity is assessed by testing the performance of reagents (e.g., primers, antibodies etc.) using a panel of isolates, the specificity of HTS methods could be assessed by verifying inclusivity and exclusivity of the database(s) of sequences used in the bioinformatic approach.

### Reproducibility and repeatability

As obtaining a diagnostic result by HTS is a multi-phasic process, the approach, platform, bioinformatic strategy, interpretation all need to be considered for reproducibility. Thus far there have been only limited investigations into the reproducibility of these various phases. Comprehensive studies testing the same sample through different approaches have not yet been performed. The comparability of different sequencing approaches has been partially investigated by Visser et al. (2016) and Pecman et al. (2017). The latter study compared several strategies (small RNA and ribosomal depleted total RNA) using viruses representing a range of genome structures and replication strategies. For known viruses, although some virus types were more efficiently detected by one or the other approach, the results of each approach were comparable. Systematic studies on repeatability and reproducibility have yet to be published, although resampling of data to explore the impact of sequencing depth indicated a high degree of reproducibility, with qualitatively different results occurring only when reducing sequence depth negatively impacted sensitivity (Visser et al., 2016; Pecman et al., 2017). This again highlighted a link between appropriate depth of coverage and the repeatability of the test.

### Additional considerations

Harnessing the diagnostic power and flexibility of HTS for screening applications also brings the inherent tension of how to deal with inadvertent non-target findings. These may be commensal or mutualistic viruses (Roossinck, 2015), but some may be known and/or unknown pathogenic viruses (Skelton et al., 2018). Such viruses may pose a risk to the tested species and/or to other potential hosts. Dealing with these findings can only be done on a case by case basis and will depend upon the virus detected and the purpose of testing (Massart et al., 2017).

For vegetatively propagated crops it is conceivable that mother plants could be accompanied by a HTS sequence metadata passport obtained according to recognized standards (Saldarelli et al., 2017). To support such advances there is a need to understand the virome of a given crop (MacDiarmid et al., 2013) and it should be understood that in some crop systems a phytosanitary declaration of ‘freedom from viruses’ may be unachievable so that a baseline of “normal” and therefore acceptable virus presence will need to be determined.

Contamination, potentially leading to erroneous reporting is recognized as a significant issue in HTS (Dickins et al., 2014). In common with other molecular techniques contamination of samples with nucleic acid from other samples can occur at a number of steps in the HTS protocols. Contamination within the platform however is a specific issue resulting from the use of “genome sequencers” for diagnostic applications. Appropriate use of negative controls during the process and introduction of cut-offs based on signal-to-noise is a solution used in many routine testing laboratories deploying other, similarly sensitive techniques. In the longer term, the advent of more diagnostic-focused platforms may improve this situation.

Use of adequate and appropriate first-line controls is essential for demonstrating that a given assay is performing within acceptable criteria, allowing for the correct interpretation of results (Roenhorst et al., 2018) but the introduction of a positive control for each target virus would introduce an unacceptable risk of contamination. To address this, two positive control strategies have been developed, using known nucleic acids as an internal control. One uses leaf discs from a plant infected with a known virus (Kesanakurti et al., 2016), whereas the other uses synthetic sequence transcripts (Jiang et al., 2011). Both strategies are considered adequate in current virus discovery applications, however this may not be the case when trying to detect a range of known viruses and other strategies, such as including a suite of non-target viruses as a positive control, could be investigated.

Alongside the appropriate use of controls, expertise in bioinformatics and plant virology has been identified as critical for the effective interpretation of diagnostic data (Roenhorst et al., 2018). The proficiency test performed in the frame of COST Action FA1407 (Massart et al., 2018) highlights significant variability in pipeline performance/expertise of users and shows that only a fraction of the laboratories were able to detect all the agents tested, while each of them was confirmed by classical RT-PCR assays.

## Conclusion

The advancement in HTS technologies undoubtedly brought great potential for virus detection and discovery. However, like any new technology, HTS-based approaches should be validated for sensitivity, specificity, reproducibility and repeatability before their routine implementation. Solutions need to be brought to deal with HTS specific challenges, such as the use of controls appropriate for the diagnostic workflow in which the method is implemented or the handling of the detection of novel or non-target viruses. Application-specific validation will ensure that the performance of HTS methods is equivalent or better than those of current targeted approaches. As more laboratories access HTS and apply it to routine virus detection there will be an increasing need for both test performance studies and regular proficiency tests to evaluate the methods themselves and the capabilities of diagnostics laboratories. Such studies will require access to a range of well characterized virus isolates and data sets and should address both the competence of the laboratory to perform all the steps of the diagnostic process, including the bioinformatics analysis, as well as its expertise in interpretation of results.

As a diagnostic tool, HTS is perhaps more broad-spectrum than any previously used assay. Whilst the technique is powerful, the available frameworks of validation (e.g., EPPO PM7/98) and diagnostic workflows (e.g., Massart et al., 2017) appear suitable to facilitate its adoption. Expanding its use to include current as well as future advancements in HTS applications requires integration and validation steps that are all well known to diagnosticians and should not be a cause for concern.

## Author contributions

All authors listed have made a substantial, direct and intellectual contribution to the work, and approved it for publication.

### Conflict of interest statement

The authors declare that the research was conducted in the absence of any commercial or financial relationships that could be construed as a potential conflict of interest.
